# Functional Validation of *cas9*/GuideRNA Constructs for Site-Directed Mutagenesis of Triticale *ABA8′OH1 loci*

**DOI:** 10.3390/ijms22137038

**Published:** 2021-06-29

**Authors:** Krzysztof Michalski, Christian Hertig, Dariusz R. Mańkowski, Jochen Kumlehn, Janusz Zimny, Anna M. Linkiewicz

**Affiliations:** 1 GMO Controlling Laboratory, Plant Biotechnology and Cytogenetics Department, Plant Breeding and Acclimatization Institute—National Research Institute, Radzików, 05-870 Błonie, Poland; krzysztof.michalski@ihar.edu.pl (K.M.); j.zimny@ihar.edu.pl (J.Z.); 2 Plant Reproductive Biology, Leibniz Institute of Plant Genetics and Crop Plant Research (IPK), 06466 Seeland, Germany; kumlehn@ipk-gatersleben.de (J.K.); hertig@ipk-gatersleben.de (C.H.); 3 Laboratory of Seed Production and Plant Breeding Economics, Department of Seed Science and Technology, Plant Breeding and Acclimatization Institute—National Research Institute, Radzików, 05-870 Błonie, Poland; d.mankowski@ihar.edu.pl; 4 Institute of Biological Sciences, Faculty of Biology and Environmental Sciences, Cardinal Stefan Wyszynski University in Warsaw, Wóycickiego 1/3 Street, 01-938 Warsaw, Poland

**Keywords:** genome editing, CRISPR, protoplasts, targeted mutagenesis, TREX2, construct validation, transient expression

## Abstract

Cas endonuclease-mediated genome editing provides a long-awaited molecular biological approach to the modification of predefined genomic target sequences in living organisms. Although *cas9*/guide (g)RNA constructs are straightforward to assemble and can be customized to target virtually any site in the plant genome, the implementation of this technology can be cumbersome, especially in species like triticale that are difficult to transform, for which only limited genome information is available and/or which carry comparatively large genomes. To cope with these challenges, we have pre-validated *cas9*/gRNA constructs (1) by frameshift restitution of a reporter gene co-introduced by ballistic DNA transfer to barley epidermis cells, and (2) via transfection in triticale protoplasts followed by either a T7E1-based cleavage assay or by deep-sequencing of target-specific PCR amplicons. For exemplification, we addressed the triticale *ABA 8′-HYDROXYLASE 1* gene, one of the putative determinants of pre-harvest sprouting of grains. We further show that in-del induction frequency in triticale can be increased by TREX2 nuclease activity, which holds true for both well- and poorly performing gRNAs. The presented results constitute a sound basis for the targeted induction of heritable modifications in triticale genes.

## 1. Introduction

Genome editing in cereals greatly facilitates genetic improvements that would have been more cumbersome with previously existing mutagenesis tools. A particularly powerful platform for site-specific changes in plant genomes is now available for which components of the microbial clustered regularly interspaced short palindromic repeats (CRISPR)/CRISPR-associated (Cas) endonuclease immune system have been repurposed [1]. These RNA-guided Cas endonucleases have been employed to generate loss-of-function alleles by targeting single or multiple *loci* in bread wheat, barley, rice, and other grass species but not yet in triticale. Up to date, reports of successful genome editing using Cas9 in monocots are limited mainly to diploid species like rice, maize, or barley [2,3]. Few reports of successful employment in hexaploid common wheat [4,5,6,7,8,9] suggest that low transformation efficiency for wheat as well as the large genome size makes it exceedingly difficult for the Cas9/gRNA complexes to find their target. Some authors also argue that gene knockout via non-homologous end joining might be inefficient in polyploids due to its genetic redundancy [10,11]. Here, we have designed a suite of constructs to examine the potential for Cas9-mediated editing in triticale that is another hexaploid species. The complexity of the triticale genome and the difficulty of monocotyledonous plant transformation present a challenge, which is reflected by limited results hitherto reported for this species. It is necessary to improve the specificity and efficiency of Cas9 on-target mutagenesis and to develop a straightforward in vivo validation system for cas9/gRNA constructs, since the generation of transgenic triticale plants is not only cost and time-consuming, but has been achieved only by very few laboratories worldwide.

Despite the great potential of the new biotechnological tools, not all *cas9*/gRNA construct designs are equally successful, which is due to several factors as reviewed by [12,13,14]. Since the Cas9 mode of action, besides its requirement of a protospacer-adjacent motif (PAM), relies on a specific interaction between gRNA and the target sequence, it is of great importance to implement particularly suitable sequences in *cas9*/gRNA constructs used for stable plant transformation [13]. Directing the Cas9 nuclease to a specific *locus* in the genome is mainly facilitated by the user-defined gRNA. Currently available web-based tools for gRNA design offer a variety of candidate target motifs for a given gene of interest [15]. Despite these in silico predictions, not every design demonstrates equal efficiency in Cas9-catalyzed cleavage. Therefore, it is advisable to pre-validate several gRNAs to identify genomic target motifs that are likely to be effectively processed. In addition, it has been demonstrated in zebrafish that some gRNAs with high in vitro predicted activity possess poor in vivo activity, suggesting the presence of factors that restrict chromatin availability and limit the mutagenesis in vivo [16]. Conditions reflecting chromatin availability are hard to ensure by testing gRNA solely based on a PCR product, which is why the utilization of in planta systems are considered a more reliable principle for gRNA validation.

In the present investigation, we report the evaluation of *cas9*/gRNA construct performance in planta based on two methods; first, a transient expression assay developed in barley [17], and second, by triticale protoplast transfection. We have included the *THREE PRIME REPAIR EXONUCLEASE 2* (*TREX2)* sequence in some of the constructs to evaluate its utility for increased mutagenesis efficiency in triticale. The biological function of TREX2 is not well understood. TREX2 is a non-processive 3′–5′ exonuclease that has multiple functions: removes 3′ mismatches form DNA [18,19] and alters replication fork stability and mutation levels in cells defective for homologous recombination [20]. TREX 2 is most closely related to TREX1 that is a component of the SET complex that degrades 3′ ends of nicked DNA during the programmed cell death pathway in mitochondrion [21].

Cas9-mediated site-directed mutagenesis was used to disrupt *TsABA8′OH1 loci* in triticale cells. The chosen target gene *TsABA8′OH1* [22,23] controls ABA catabolic degradation in grains and is present in triticale as a single copy gene on each of its three group 6 chromosomes. Target sites on chromosomes 6A, 6B of the wheat reference genome and on chromosome 6R of the rye reference genome were selected for the conducted analyses.

## 2. Results

### 2.1. Selection of Target Motifs

The availability of triticale genomic sequence data is limited. Therefore, genomic sequences of *ABA8′OH1* of the A and B subgenomes of bread wheat (GenBank accession no. AB714574.1 and AB714575.1, respectively) and a contig containing the putative rye homolog from the USDA database (Lo7_v2_contig_2868507) were used to design common primers for the simultaneous amplification of all three homeologues of the target gene (Figure S1).

**Figure 1:**
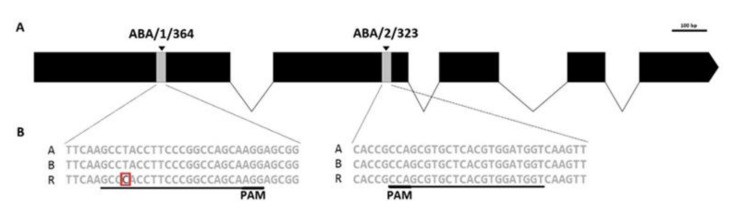
(**A**) Schematic diagram of target ABA8′OH1 regions used for selection of two gRNAs. Black boxes indicate exons, black lines indicate introns, gray boxes represent the regions in exon 1 and exon 2 designated for Cas9/gRNA-induced mutagenesis. (**B**) Detailed sequence context of target motifs (underlined, with PAM indicated) on A, B, and R of triticale sub-genomes. Notably, one SNP is present in the target motif ABA/1/364 of the R genome.

To uncover potential nucleotide polymorphisms between these data and the triticale subgenomes, which may affect targeted mutagenesis, the first two exons of *TsABA8′OH1* from the Polish winter triticale variety Bogo, an accession that has been used as experimental model for genetic engineering, were amplified using common primers (Table S1), then cloned in plasmids and sequenced (Table S1). The resulting genomic comparison of *TsABA8′OH1* sequences from cv. Bogo (NCBI MW538321-MW538323) showed that the percentage in identity of these regions to the reference sequences ranged from 98.7% for the R genome to 100% for the B genome (Figure 1). By contrast, essential differences were seen within the introns of the *TsABA8′OH-1* homoeologues, which facilitated the design of subgenome-specific primers for PCR amplification (Figure S1).

### 2.2. Utility of Cas9/gRNA Target Motifs in ABA8′OH-1 of Triticale

**Figure 2:**
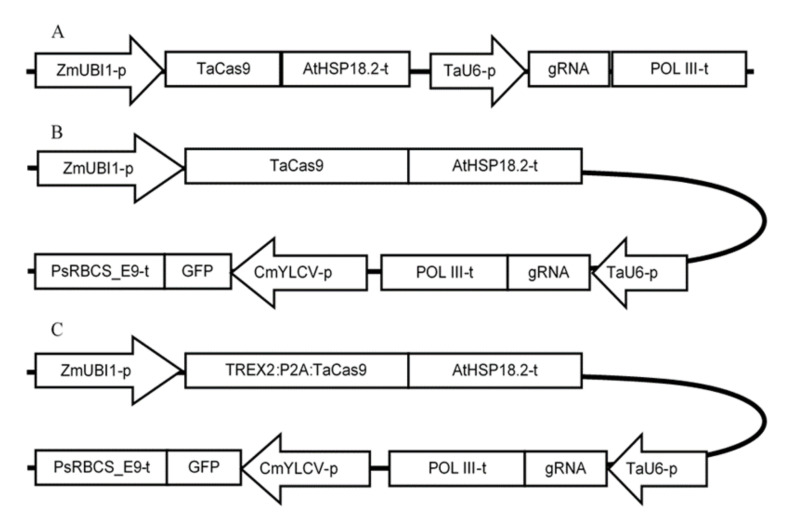
Schematic illustration of the *cas9* and gRNAs expression cassettes in (**A**) the transient expression vector used for the barley transformation experiment, and a binary vectors lacking (**B**) or containing (**C**) TREX2 nuclease sequence used for protoplast transformation. TaU6-p—*Triticum aestivum U6* promoter, POL III-t—*RNA POLYMERASE III* terminator, CmYLCV-p—strong constitutive promoter from *Cestrum yellow leaf curling virus*, GFP—sequence of *GREEN FLUORESCENT PROTEIN* gene, ZmUBI1-p—Zea mays *POLYUBIQUITIN 1* promoter, gRNA—single guide RNA scaffold, PsRBCS_E9-t—pea *RIBULOSE BISPHOSPHATE CARBOXYLASE SMALL SUBUNIT* terminator, TaCas9—*Triticum aestivum* codon-optimized *CRISPR-associated protein 9*, AtHSP18.2-t—*Arabidopsis thaliana HEAT SHOCK PROTEIN 18.2* terminator, TREX2—monocot codon-optimized human *THREE PRIME REPAIR EXONUCLEASE* 2, P2A—*Porcine teschovirus-1 2A* self-cleaving peptide motif.

Three different approaches were pursued to evaluate the editing activity of Cas9/gRNA customized for two target motifs in the *ABA8′OH-1* gene of triticale. This involved the computational prediction of genomic sequences suitable to Cas9-generated double strand breaks (DSBs) and off-target site prediction in wheat and rye genomes. In the second step, *cas9*/gRNA constructs were evaluated in planta as previously described by Budhagatapalli et al. [17]. In this method, the mutation activity of customized endonuclease constructs (Figure 2A) is estimated based on the restitution of a reporter gene upon ballistic DNA transfer to barley epidermis cells. In addition, Cas9/gRNA activity was validated at the sequence level via triticale protoplast transfection followed by amplification of target regions and sequencing.

### 2.3. Selection of Target Motifs under Consideration of Predicted On- and Off-Targets

Two gRNAs (gRNA-ABA/1/364 and gRNA-ABA/2/323) were selected for gene editing based on their in silico predicted on-target activity and PAM presence, whereby the scores for gRNAs ranged from 51.3 to 60.9 according to the Cas-Designer online tool [24]. gRNA-ABA/1/364 and gRNA-ABA/2/323 were chosen to target all triticale homeoalleles of *TsABA8′OH1*, with the gRNA-ABA/1/364-addressed target motif containing a single mismatch 17 nt upstream of the PAM site in the R genome (Figure 1). 

It is essential to ensure that the gRNA sequences match their cognate target *loci*, but do not match additional sites within the genome. Off-targets predicted by the Cas-Designer tool and a simple BLAST of the wheat and rye reference genomes featured at least two mismatches in comparison to their on-target counterparts (Table 1).

### 2.4. Modeling the Secondary Structure of Guide RNAs

**Figure 3:**
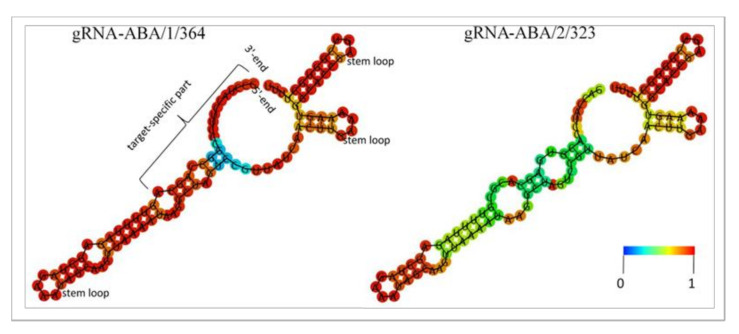
Secondary structure models of guide RNAs. Two-dimensional models of the minimum free energy structures of gRNA-ABA/1/364 and gRNA-ABA/2/323 as generated by the RNAfold online platform (http://rna.tbi.univie.ac.at/cgi-bin/RNAWebSuite/RNAfold.cgi (accessed on 25 April 2021)) [26]. RNA ends, target-specific part and essential stem loops are indicated only for gRNA-ABA/1/364 but apply likewise for the other as well. The color code represents the base-pairing probabilities of individual nucleobases. For unpaired bases, the colors denote the probabilities of being unpaired.

In addition to the target motif-specific 3′ region, the gRNAs comprise a scaffold that is required for the formation of ribonucleoprotein complexes with Cas9 endonuclease. Three stem loop structures are essential for binding with the Cas9 protein. These stem-loops result from short self-complementary inverted sequence repeats within the gRNA scaffold [25]. Depending on its sequence, the target motif-specific region of the gRNA can interfere with the formation of these stem loops by binding complementary bases. Figure 3 shows that the 2D models of both tested gRNAs feature the three functionally essential stem loops and should therefore be well-suited for Cas9-mediated mutagenesis. This analysis further revealed that the frequency of the calculated gRNA-ABA/1/364 and gRNA-ABA/2/323 structures within their ensembles of variants is 4.4% and 6.6%, respectively.

### 2.5. Assessment of Gene Editing Frequency via Frameshift Restitution in a Transiently Expressed Reporter Gene

We have cloned synthetic target regions of *ABA8′OH-1* into *Bam*HI and *Eco*RI sites of pNB1 (pTARGET) vector [17]. The performed transient expression test indicates cleavage activity via frameshift restitution of a *YFP* reporter gene. Barley leaves were co-bombarded with ABA8′OH-1_pTARGET vectors, *mCHERRY* vector, and vectors encoding specific gRNAs and Cas9. Construct activity was calculated as the proportion of cells with restored YFP functionality among all transformed cells showing mCHERRY signal [17]. Both gRNA-ABA/1/364 and gRNA-ABA/2/323 showed detectable activity and restored YFP functionality when the *cas9*/gRNA constructs were co-transfected with the *mCHERRY* reporter construct (Figure S3). While gRNA-ABA/1/364 yielded only 5.6% of YFP-positive cells among all mCHERRY expressing cells in barley, gRNA-ABA/2/323 led to *YFP*-frameshift repair cells in 24.3% of transfected cells on average (Table 2).

### 2.6. Assessment of Gene Editing Frequency in Triticale Protoplasts Using T7E1 Assay

Here, we have adopted a protocol of wheat protoplast transfection [27] for triticale and optimized the process of protoplast isolation and transfection in the latter species. In order to validate the functionality of assembled gRNA expression units (Figure 2B,C) and to assess their efficiency in targeted mutagenesis, triticale protoplasts were transfected with customized *cas9*/gRNA constructs using PEG-mediated DNA transfer. Transfection efficiency was evaluated based on *GREEN FLUORESCENT PROTEIN* (*GFP*) expression in the protoplasts (Figure S2). The highest transient expression efficiency (ranging from 40% to 60%) was achieved with a plasmid concentration of 25 µg per 100,000 cells and the density of protoplasts being adjusted to 2.5 × 10^5^ cells/mL prior to adding PEG.

To see if we can enhance the efficiency of mutagenesis for gRNA-ABA/1/364 and gRNA-ABA/2/323, *TREX2*-enhanced vectors (+TREX2) [28] were used for comparison with the conventional constructs (Table 3 and Table S3A). 

A *T7 endonuclease I* (T7E1) assay [29] was used to detect mutation events in the target motifs of transfected versus untreated protoplasts. T7E1 results confirmed that the used constructs were capable of inducing mutations in their cognate target motifs in vivo. We observed that the addition of the *TREX2* sequence in the constructs generally increased the editing efficiency. The TREX2-mediated improvement in editing efficiency was significant in the case of the triticale A genome addressed by gRNA-ABA/1/364, where the increase was about 3.3 times (*p* < 0.0001). Even higher differences were observed for gRNA-ABA/2/323 in the comparison of no-*TREX2* and TREX2+, where the editing efficiency in the R genome jumped from an undetectable level to 28.1% (Table 3). Regarding no-*TREX2* constructs, the gRNA-ABA/1/364 was observed to be the most active on the R genome locus, with the mean indel frequency being 24.5%, followed by the B (15.6%) and A genome loci (6.1%) (*p* = 0.01). A significantly lower number of edited sequences on A, B, and R genomes were observed for no-*TREX2* gRNA-ABA/2/323 construct (*p* < 0.0001) (Table 3).

### 2.7. Assessment of Targeted Mutagenesis Frequency in Triticale Protoplasts Using Deep-Sequencing of Short Reads

To assess the quantity and pattern of induced mutations with or without *TREX2* in the constructs and to crosscheck the results from T7E1 analysis, PCR products amplified from the target regions of transfected protoplast-derived genomic DNA were subjected to deep-sequencing. The analysis of reads was conducted using the Geneious Prime Software by which we were able to correctly map the reads to the respective reference sequences of A, B, or R genomes. On average, about 368,000 high quality reads were generated for each sample. The total number of reads assigned to target motifs 1 and 2 on A, B, and R genomes ranged from about 15,000 to 135,000.

**Figure 4:**
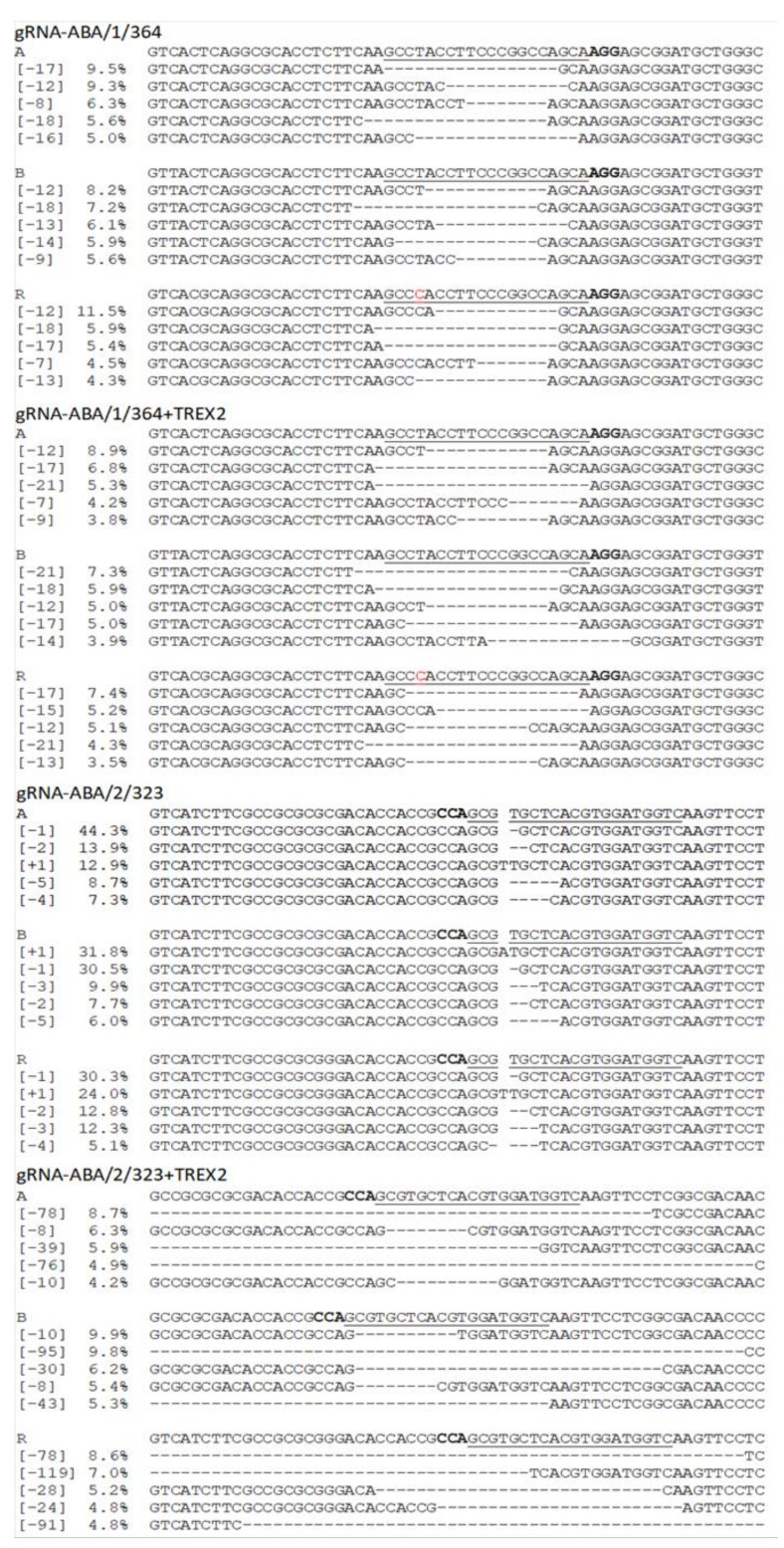
Relative frequency of the most abundant mutations among changes induced on each subgenome of triticale protoplast by transient expression of cas9/gRNA-ABA/1/364 and cas9/gRNA-ABA/2/323 constructs, lacking or containing TREX2 enhancer. PAM motif bolded; target motif underlined. SNP present in the target motif ABA/1/364 of the R genome is marked in red.

We detected modifications in the target regions in all samples (Table 4), except for the negative controls, where modifications were not detectable beyond the level of sequencing errors. Deletions were the predominant type of occurring mutations, while some insertions and substitutions were also observed. The most abundant modifications in regions targeted by gRNA-ABA/1/364 and gRNA-ABA/2/323 vectors equipped with *TREX2* or not, are summarized in Table S2. We observed high variability in mutagenesis efficiency and generated mutation types between the two target motifs addressed, with mainly 11–99 bp or 2–10 bp deletions being detected in case of target ABA/1/364, and 2–10 bp deletions together with 1 to 99 bp insertions typically seen in target ABA/2/323 (Figure 4). In the experiment where no *TREX2* vectors were applied, the total efficiencies of identified modifications ranged from 2.0% for gRNA-ABA/2/323 to 30.4% in case of gRNA-ABA/1/364, with both of these values derived from the respective target motifs of the A genome.

We have noticed an increased mutation frequency in case of both gRNAs when the *cas9* gene was supplemented with the *TREX2* sequence, which resulted in nearly 1.7-fold increase of total mutagenesis efficiency for gRNA-ABA/1/364 and nearly 15-fold for gRNA-ABA/2/323. Notably, TREX2 activity significantly reduced the proportion of single nucleotide deletions for gRNA-ABA/2/323 and small (2–10 nt) deletions for gRNA-ABA/1/364 constructs, simultaneously increasing frequencies of medium (11–99 nt) and large (>100 nt) deletions in both gRNAs tested. Additionally, TREX2 significantly reduced the occurrence of medium-size insertions (Tables S2 and S3B).

Regardless of the constructs used, we did not observed statistically significant differences in mutagenesis frequencies between the analyzed sub-genomes (Table 4 and Table S3C).

## 3. Discussion

Estimation of Cas9/gRNA activity by computational methods may not be accurate for all applications as the criteria are derived from context-specific data [30]. Available methods for the analysis of on-target and off-target mutations have some limitations and some benefits that were comprehensively discussed by Zischewski et al. [31]. The ideal methods should take into account the particular genome edited, anticipated size, and type of mutation as well as cost of the method. Thus an experimental validation of any given gRNA activity is advisable. It is highly beneficial to determine which constructs have the highest potential for successful genome editing, before starting with the cumbersome methods of genetic engineering at the whole-plant level. To date, quick and efficient transformation systems are still not available for some plant species, including triticale. Therefore, protoplast transfection or transient transgene expression in other cell systems are useful validation tools for multiple mutagenesis parameters.

*cas9*/gRNA constructs can exhibit a broad range of efficiencies. Testing multiple gRNAs increases the chance of identifying a construct that is specific and highly active [32,33]. Several vector sets have been developed for genome editing in plants [28,34,35,36,37]. We have decided to use a multiplasmid, flexible system developed by Čermák et al. [28] available from plasmid repository ADDGENE, that enables targeted, specific modification of monocot genomes, because its utility has been demonstrated by several authors [14,15,38]. To verify whether multiple homeoalleles of the *ABA8’OH-1* gene can be simultaneously addressed, we were taking into account the nature of individual target sites, including PAM presence, gRNAs design, possibility of off-target activity, and two different construct variants.

Cas9 has been shown to preferentially binding DNA targets with purines in the four PAM-proximal bases of the targeting sequence, whereas pyrimidines and, especially, thymines were disfavored [39,40]. Working with human cells, Graf et al. [41] discovered that TT-motif and GCC-motifs within the four PAM-proximal bases of the target motif negatively impact the efficiency of gRNAs resulting in a about 10-fold reduction in editing frequency. The gRNAs used in the present investigation do not contain any TT-motifs. On the other hand, the four base PAM-proximal region of gRNA-ABA/1/364 contains three purines, whereas in gRNA-ABA/2/323 there are two. To find out whether these features have an impact on the formation and function of Cas9/gRNA complexes is beyond the scope of this study.

### 3.1. Off-Target and On-Target SNP Evaluation

*cas9*/gRNA constructs typically include a 20-bp gRNA 5′ end that directs the Cas9 nuclease to the target site by complementary base pairing. It is well established that the gRNA sequence plays a pivotal role in determining the on- and off-target activities of Cas9 [42,43]. Moreover, in silico analysis of gRNA sequences are only approximations to the experimental data. In general, off-target sites are not cleaved as efficiently in particular when mismatches occur near the PAM, so gRNAs with no homology or those with mismatches close to the PAM sequence will have the highest specificity [44]. Our results show that the closest sequence to the targets of gRNA-ABA/1/364 and gRNA-ABA/2/323 has at least two mismatches within the 20 bp region bound by the cognate gRNA. However, potential off-target matches on the 6R for gRNA-ABA/1/364 and on the 5A/5B/5R for gRNA-ABA/2/323 should be evaluated more carefully than the others, as the SNPs are not located in proximity to the PAM.

We have found common target motifs on A, B, and R homeologous triticale genomes in exon 1 and 2 of *ABA8′OH-1*, having only one mismatch for the gRNA-ABA/1/364 that might influence the processing of the R genome. However, the R genome was as efficiently mutated as those derived from wheat. A possible explanation is that the SNP position is outside the core region of the gRNA-ABA/1/364 target sequence [44].

### 3.2. Protoplast Transfection as a Convenient Test for Cas9/gRNA Functionality

In the present study, we assessed and verified the activity of two gRNAs targeting the *ABA8′OH-1* gene using protoplast transfection and transient expression in the barley leaf epidermis. The major advantage of protoplast transfection followed by amplicon sequencing, as compared to the expression of test constructs in epidermis cells, is that the Cas9/gRNA target motifs are processed in their chromosomal context. Protoplasts from model species such as *Arabidopsis* and tobacco as well as crops like rapeseed, rice, wheat, and maize have been used to evaluate reagents of Cas9-based systems [27,45,46,47,48]. However, this method requires experience in terms of handling delicate cell cultures and a careful optimization of conditions depending on the species used. Here, we used a protoplast isolation and transfection protocol previously available for wheat, adapted it for triticale and eventually achieved Cas9/gRNA-triggered mutagenesis with useful efficiency.

Methods like deep-sequencing of PCR amplicons or the T7E1 cleavage assay are available to assess targeted mutagenesis. In the present study, both approaches were applied and compared. The T7E1 assay is widely used, cost-effective, technically straightforward, and easy to interpret. However, Vouillot et al. [29] revealed that 1 bp indels are not recognized by this method, which is why it is likely to result in underestimations of the total mutagenesis efficiency. In line with this, we were not able to detect any modification in the R genome and only very few on the A and B sub-genomes after transfection of the construct harboring the no-*TREX2* gRNA-ABA/2/323. By contrast, the deep-sequencing data revealed 2.0–3.3% total mutagenesis efficiency when using the same construct, coming mostly from 1 bp and 2–10 bp deletions and small insertions. No such bias between T7E1 and amplicon sequencing has been observed when TREX2+ constructs were used.

While most of the observed mutations in protoplasts were deletions and single nucleotide substitutions, insertions were only rarely found. The mutation efficiency demonstrated by deep-sequencing was as high as 48.8%. This result suggests that our best-performing construct may also be useful at the whole-plant level.

### 3.3. Gene Editing Frequency in Plants Can Be Estimated via Transient Expression

A biolistic-based transient expression test for *cas9*/gRNA constructs using leaf epidermis was previously established and used in barley [17,49]. Here, we were able to employ this method for evaluation of no-*TREX2* constructs through co-expression with triticale-derived targets in barley leaves. Both, gRNA-ABA/1/364 and gRNA-ABA/2/323 showed acceptable efficiency in this test, which is contradictory to the results obtained in triticale protoplasts by deep-sequencing, where gRNA-ABA/2/323 no-*TREX2* construct showed very poor activity. Possible reasons for such inconsistent results might be that not only the different species—barley and triticale, but also different cell types were used, in which DNA repair underlie specific circumstances. Moreover, the non-genomic nature of the target plasmids co-introduced into barley epidermis cells is likely to be of some importance. Despite the observed discrepancies, the deep-sequencing analysis strongly suggests that this approach is more suited and accurate for the detection of small indels. It is within reason to suspect that smaller insertions and deletions are more likely to repair the *YFP* reading frame as it happened in case of gRNA-ABA/2/323. Meanwhile, the larger indels, even when correcting the frame-shift, might cause some unwanted changes to YFP protein activity.

### 3.4. The Effect of TREX2 Co-Expression on Cas9-Mediated Gene Editing

Some additional strategies can be pursued to further improving the editing efficiency of *cas9*/gRNA constructs. To increase the mutagenesis efficiency achieved by Cas9, we have tested the TREX2 enhancer for its utility as a transcriptionally fused sequence [50,51]. TREX2 was reported to increase frequencies of heritable mutations in tomato and barley protoplasts by 2.5-fold [28]. Similarly, targeted mutagenesis efficiency was further improved by TREX2 in *Setaria viridis* by an average of 1.4-fold [38]. The *TREX2* gene encodes a nuclear protein with 3′ to 5′ exonuclease activity. The encoded protein participates in double-stranded DNA break repair [52]. TREX2 does increase mutation rates across both on- and off-target sites to varying degrees. Based on the work of Zuo and Liu [50], we assume that Cas9 can generate during the DNA cleavage process 1-bp overhangs on the nontarget strand (ntDNA), leaving short staggered ends. Here, we see the possible role of TREX2 in removing 3′ overhangs on the ntDNA leaving larger gaps for the cell repair mechanism and making whole process more cumbersome.

We have found that the TREX2 significantly increased editing efficiency in triticale even for the low performing gRNA-ABA/2/323, which was equally clear in both T7E1 and deep-sequencing assays. In the latter, it was observed that *TREX2*-including vectors caused an increased occurrence of >10 bp deletions, while reducing small (1–10 bp) deletion and insertions. This observation is in accord with the results of Čermak et al. [28].

### 3.5. Prospects of Triticale Cas9-Mediated Gene Editing

Recent advances in genome editing techniques demonstrate the potential to accelerate breeding of triticale for preharvest sprouting resistance (PHS) and disease resistance. Cas endonuclease-mediated genome editing has emerged as the most powerful tool for crop improvement due to its design simplicity and capability convenient generating alterations in plant genome. This article presents the first report of CRISPR/Cas9-based genome editing of triticale sequences with improved effectivity due to the use of modified vectors. Protoplast transfection or transient transgene expression in barley cell systems can be seen as a useful validation tools for multiple mutagenesis parameters due to the lack of an efficient and easy transformation systems for triticale. Presented gRNA constructs targeting *TsABA8′OH1* gene may be used for stable modification of triticale plants more resistant to PHS. However, the current status of regulatory requirements for the release of genome-edited crop in the EU may slow down the application from practical use for breeding purpose.

## 4. Materials and Methods

### 4.1. Plant Material and ABA8′OH-1 Identification in Triticale

Triticale cv. Bogo (EGISET, Accession Number: 66000), a highly regenerable hexaploid triticale cultivar, was used as a material source in all described experiments. If not stated otherwise, 7 days-old plants were vernalized for 8 weeks at 4 °C and then cultivated under controlled conditions in a growth chamber with 16 h photoperiod and 21 °C temperature.

Genomic sequences of *ABA8′OH-1* genes corresponding to both A and B genomes of bread wheat were verified and downloaded from the National Center for Biotechnology Information (NCBI). Wheat sequences were used to find scaffolds containing rye homologs in the USDA database. Homologous sequences of A, B, and R genomes were aligned and conserved regions in exon 1 and exon 3 were used to design common primer pairs (Figure S1) [53,54].

DNA isolated from fresh leaves was used as a template in PCRs. Reaction products were cloned directly into pJET2.1 (CloneJET PCR Cloning Kit; Thermo Fisher Scientific, Waltham, MA, USA) vector and transformed into *E. coli* strain DH5α with CaCl_2_/heat shock method. Twenty single colonies were used for plasmid isolation with Qiagen MiniPrep Kit (Qiagen; Hilden, Germany) and sent for Sanger sequencing.

### 4.2. gRNA Design, 2D Modeling, and Vectors Construction

Cas-Designer on-line tool [24] was used to design gRNA candidates based on wheat A genome sequence and to predict the specificity of target to B and R genomes based on bread wheat genome (assembly: IWGSC1.0, 2014) and rye contig (Lo7_v2_contig_2868507). Only targets adjacent to an appropriate protospacer adjacent motif (PAM) for Cas9 (NGG) were considered. For the purpose of the study and based on the specificity to each and every triticale genomes, two gRNA were selected for the gene and named gRNA-ABA/1/364 and gRNA-ABA/2/323, thereby indicating the addressed exons (1 or 2) and the nucleotide positions within the gene. To assess the formation of the secondary structures of the gRNAs, the two preselected gRNAs were subjected to 2-dimensional modeling for which the online tool RNAfold (http://rna.tbi.univie.ac.at/cgi-bin/RNAWebSuite/RNAfold.cgi (accessed on 25 April 2021)) was used [26]. This tool determines and displays the minimum free energy structural variant of a given RNA sequence, i.e., the one among the many possible structures of an RNA that has the highest stability and thus is present with the highest abundance.

Plasmids developed by the Voytas Lab [28] were used for two-step, GoldenGate-based construction of expression vectors. This plasmid series provided starting material to generate a RNA-guided Cas9 tool for gene editing in plants. At first, synthetic oligonucleotides, ordered externally, were annealed and cloned into pMOD_B vectors. In the second step, Cas9 or Cas9 + TREX2-coding pMOD_A plasmids were mixed with pMOD_B and pMOD_C plasmids and cloned into pTRANS expression vectors. Different pMOD_A, pMOD_C and pTRANS plasmids were used, depending on downstream application.

### 4.3. Transient Expression Method

Synthetic oligos coding target sequences were introduced into pNB1 vector causing non-sense frameshift in *YELLOW FLUORESCENCE PROTEIN* (*YFP*) gene [17]. Thus, acquired pTARGET vectors were mixed with mCHERRY-coding pmCHERRY and *cas9*/gRNA vectors and coated onto 1 µm gold particles. BioRad PDS-1000 system was used for bombardment of leaves taken from 10-days old barley cv. Golden Promise seedlings. Plasmids used for this assay contained only *cas9* and gRNA cassettes with no *TREX2* and GFP coding sequences. Mixes of empty (with no frameshift in *YFP*) pNB1 and pmCHERRY, as well as pmCHERRY and one of pTARGET vectors were used as positive and negative controls, respectively. After 48 h incubation, leaf fragments were fixed to glass slides and subjected to observation in Zeiss LSM780 confocal laser microscope. Cas9/gRNA activity was calculated as the ratio between the number of cells with restored YFP signal compared to cells with mCHERRY signal.

### 4.4. Protoplast Transfection Assay

Methodology previously optimized for wheat protoplasts transfection [27] was adapted for triticale. Triticale protoplasts were transfected with preassembled vectors via polyethylene glycol (PEG)-mediated delivery. Seven days-old, etiolated seedlings of triticale cv. Bogo were used for protoplast isolation. The central part of the second leaves were chopped with new razor blades and incubated for 5 h in W5 containing cellulase R-10 (Duchefa) and macerozyme R-10 (Duchefa) at 25 °C. Protoplasts were then collected and immediately transfected with 25 µg of plasmid vector per 100 thousand cells. The four vectors were transfected using PEG treatment into triticale protoplasts. After 48 h incubation in darkness, at room temperature, without shaking, transfection efficiency was evaluated based on *GREEN FLUORESCENT PROTEIN* (GFP) expression. Genomic DNA was extracted from transfected protoplasts and used for PCR. Fragments of the *ABA′OH-1* targeted sequences were amplified using PCR with the specific primers (Table S1), cleaned-up, re-annealed, and digested with T7 endonuclease. Digestion products were visualized with BioAnalyzer 2100 using High Sensitivity DNA chips. Cas9/gRNA activity was calculated based on the ratio of digested and undigested products.

### 4.5. Deep-Sequencing of Amplicons

Genomic DNA isolated previously from transfected protoplast was used to amplify target regions with primers containing adaptors for subsequent NGS library synthesis. Library synthesis and Illumina HiSeq 2000 sequencing were conducted by a third party company. Thus, acquired reads were then trimmed and mapped to reference sequences with the Geneious Prime (v2020.2.4) software [55]. Finally, a build-in tool was used to count Cas9-induced modification frequencies in sequences annotated to particular genomes.

### 4.6. Statistical Analysis

In order to assess the differences in editing efficiency between constructs, a series of one-way analyses of variance were performed together with the Tukey method for multiple comparison procedure. Analyses were performed for all data combined and divided by each genome. For the variance analysis Bliss transformation was applied on the data and the Statistica program ver. 13.3 (TIBCO Software, Inc., 2017, Palo Alto, CA, USA) was used [56].

## Figures and Tables

**Table 1:** Polymorphisms between on-target motifs and potential off-targets of the cas9/gRNA constructs used in this study. Predicted specificity of cas9/gRNA editing in triticale based on common wheat and rye genome induced off-target events. PAM sequence is underlined, mismatches as opposed to the target are indicated by red letters.

Chromosome	Species	Sequence 5′-3′	Target
6A/6B	triticale	GCCTACCTTCCCGGCCAGCAAGG	On-target motif ABA/1/364
6R	triticale	GCCCACCTTCCCGGCCAGCAAGG	On-target motif ABA/1/364
6R	rye	GCCGACGTTCCCGGCCAGCAAGG	Potential off-target motif ABA/1/364
7B	wheat	GCATACCTTCCCG-CCAGCATGG	Potential off-target motif ABA/1/364
6A/6B/6R	triticale	GACCATCCACGTGAGCACGCTGG	On-target motif ABA/2/323
3A	wheat	GACCATCCACGTGAG-ACTCAGG	Potential off-target motif ABA/2/323
3B	wheat	GACCAGCC-CGTGAGCACGCCGG	Potential off-target motif ABA/2/323
4A	wheat	G-CCAGCCACGTGAGCACGCCGG	Potential off-target motif ABA/2/323
5A/5B/5R	wheat, rye	GACGATCCAGGTGAGCACGCTGG	Potential off-target motif ABA/2/323

**Table 2:** Relative cleavage activity upon transient expression of cas9/gRNA and YFP/mCHERRY test constructs in barley leaf epidermis. Mean editing efficiency values ± SD is indicated in bold letters.

	Number of Red Fluorescing Cells	Number of Yellow Fluorescing Cells	Proportion of Yellow out of Total Fluorescing Cells
gRNA-ABA/1/364	33	2	6.1%
	110	6	5.5%
	38	2	5.3%
	**5.6 ± 0.42%**		
gRNA-ABA/2/323	62	14	22.6%
	101	27	26.7%
	72	17	23.6%
	**24.3 ± 0.42%**		

**Table 3:** Gene editing efficiency for gRNA-ABA/1/364 and gRNA-ABA/2/323 vectors containing or lacking the TREX2 component, analyzed in transfected triticale protoplasts using T7E1 assay. Mean editing efficiency values ± SD indicated in bold letters. ND—not determined.

Editing Efficiency [%]			
	Genome A	Genome B	Genome R
gRNA-ABA/1/364	6.3	17.1	27.3
	6.8	11.1	13.9
	5.3	18.6	32.3
	**6.2 ± 0.8 ^a^**	**15.6 ± 4.0 ^a,b^**	**24.4 ± 9.5 ^b^**
gRNA-ABA/1/364 +TREX2	14.9	35.2	32.7
	27.5	22.0	22.5
	17.8	38.9	14.1
	**20.1 ± 6.6 ^a^**	**32.0 ± 8.9 ^a^**	**23.1 ± 9.3 ^a^**
gRNA-ABA/2/323	0.3	1.9	ND
	ND	1.4	ND
	0.4	1.1	ND
	**0.2 ± 0.2 ^a^**	**1.4 ± 0.4 ^b^**	**ND**
gRNA-ABA/2/323 +TREX2	26.2	35.4	22.2
	15.6	19.2	34.7
	27.5	19.0	27.3
	**23.1 ± 6.5 ^a^**	**24.5 ± 9.4 ^a^**	**28.1 ± 6.3 ^a^**

**Table 4:** Gene editing efficiency for gRNA-ABA/1/364 and gRNA-ABA/2/323 vectors containing or lacking the TREX2 component, analyzed in transfected triticale protoplasts using amplicon deep-sequencing. Mean editing efficiency values ± SD are indicated in bold letters.

Editing Efficiency [%]			
	Genome A	Genome B	Genome R
gRNA-ABA/1/364	25.6	33.2	32.0
	27.6	20.7	23.6
	26.8	37.3	31.7
	**26.7 ± 1.0**	**30.4 ± 8.6**	**29.1 ± 4.8**
gRNA-ABA/1/364 +TREX2	50.4	36.7	38.6
	63.4	48.4	62.9
	46.7	43.5	44.8
	**53.5 ± 8.7**	**42.9 ± 5.9**	**48.8 ± 12.6**
gRNA-ABA/2/323	0.4	0.2	1.7
	2.4	2.4	5.5
	3.3	4.4	2.6
	**2.0 ± 1.5**	**2.3 ± 2.1**	**3.3 ± 2.0**
gRNA-ABA/2/323 +TREX2	31.2	50.7	43.2
	15.2	33.0	22.8
	45.9	48.9	49.8
	**30.7 ± 15.3**	**44.2 ± 9.7**	**38.6 ± 14.1**

## Supplementary Materials

The following are available online at https://www.mdpi.com/article/10.3390/ijms22137038/s1, Figure S1: TsABA8′OH partial sequences of triticale cv. Bogo generated in the study. Figure S2: Population of triticale mesophyll protoplasts transfected with CRISPR/Cas9 coding plasmid after 48 h of incubation. Figure S3: Barley leaf transient expression assay. Table S1: Sequence of primers used for PCR and sequencing. Table S2: Summarized deep-sequencing data showing the share of distinct mutation types in the A, B, and R triticale sub-genomes generated through protoplast transfection with four tested constructs. Table S3: Editing efficiency of constructs, based on one-way analyses of variance performed together with the Tukey method for multiple comparison.
