# Supplementary material for: Functional Validation of cas9/GuideRNA Constructs for Site-Directed Mutagenesis of Triticale ABA8′OH1 loci
Source: Int J Mol Sci. 2021 Jun 29;22(13):7038. doi: 10.3390/ijms22137038 (PMC8269138; doi:10.3390/ijms22137038)
Supplement: Supplementary file 1 [file ijms-22-07038-s001.zip › ijms-1240053-supplementary/Figure S1.pdf]

```
                          cov    pid   1 [         .         .         .         .         :         .         .         . 80
1 AB714574.1[genA_ex1-3] 100.0% 100.0%    ATGGCTGCTTTCGTTCTCTTCCTCTGCTTGCTCGTGCCGCTCGTCCTCGCGTGCGCCGTCCGCGCCAGGAAGAAGGGCGC
2 TsABA8'OH-A             77.9%  99.9%    --------------------------------------------------------------------------------
3 TsABA8'OH-B             76.2%  94.3%    --------------------------------------------------------------------------------
4 TsABA8'OH-R             77.0%  92.3%    --------------------------------------------------------------------------------

                          cov    pid  81           .         1         .         .         .         .         :         . 160
1 AB714574.1[genA_ex1-3] 100.0% 100.0%    CGGCGGGAGGTCGTCGTCGGGCGGCGGCGGCGGCAAGAAGGGCGGCAGCAGCAGCCTGCCGCTGCCGCCGGGGTCCATGG
2 TsABA8'OH-A             77.9%  99.9%    --------------------------------------------------------------------------------
3 TsABA8'OH-B             76.2%  94.3%    --------------------------------------------------------------------------------
4 TsABA8'OH-R             77.0%  92.3%    --------------------------------------------------------------------------------

                          cov    pid 161           .         .         .         2         .         .         .         . 240
1 AB714574.1[genA_ex1-3] 100.0% 100.0%    GGTGGCCGTACGTGGGCGAGACCACGCAGCTCTACTCCTCCAAGAACCCCAACGTCTTCTTCGCCAGGAAGCGCAACAAG
2 TsABA8'OH-A             77.9%  99.9%    -------------------------------------------------------------------------------G
3 TsABA8'OH-B             76.2%  94.3%    -------------------------------------------------------------------------------G
4 TsABA8'OH-R             77.0%  92.3%    -------------------------------------------------------------------------------G
                                                                           ABA_uF          ABA2_ngsF

                          cov    pid 241           :         .         .         .         .         3         .         . 320
1 AB714574.1[genA_ex1-3] 100.0% 100.0%    TACGGGCCCATCTTCAAGACGCACATCCTCGGGTGCCCCTGCGTCATGGTGTCCAGCCCCGAGGCCGCCAAGTTCGTGCT
2 TsABA8'OH-A             77.9%  99.9%    TACGGGCCCATCTTCAAGACGCACATCCTCGGGTGCCCCTGCGTCATGGTGTCCAGCCCCGAGGCCGCCAAGTTCGTGCT
3 TsABA8'OH-B             76.2%  94.3%    TACGGGCCCATCTTCAAGACGCATATCCTCGGGTGCCCCTGCGTCATGGTGTCCAGCCCCGAGGCCGCGAAGTTCGTGCT
4 TsABA8'OH-R             77.0%  92.3%    TACGGGCCCATCTTCAAGACGCACATCCTGGGGTGCCCCTGCGTGATGGTGTCCAGCCCGGAGGCCGCCAAGTTCGTGCT
                                              RABex1F

                          cov    pid 321           .         .         :         .         .         .         .         4 400
1 AB714574.1[genA_ex1-3] 100.0% 100.0%    CGTCACTCAGGCGCACCTCTTCAAGCCTACCTTCCCGGCCAGCAAGGAGCGGATGCTGGGCCCCCAGGCCATCTTCTTCC
2 TsABA8'OH-A             77.9%  99.9%    CGTCACTCAGGCGCACCTCTTCAAGCCTACCTTCCCGGCCAGCAAGGAGCGGATGCTGGGCCCCCAGGCCATCTTCTTCC
3 TsABA8'OH-B             76.2%  94.3%    TGTTACTCAGGCGCACCTCTTCAAGCCTACCTTCCCGGCCAGCAAGGAGCGGATGCTGGGTCCCCAGGCCATCTTCTTCC
4 TsABA8'OH-R             77.0%  92.3%    CGTCACGCAGGCGCACCTCTTCAAGCCCACCTTCCCGGCCAGCAAGGAGCGGATGCTGGGCCCCCAGGCCATCTTCTTCC
                                                               ABA/1/364      PAM

                          cov    pid 401           .         .         .         .         :         .         .         . 480
1 AB714574.1[genA_ex1-3] 100.0% 100.0%    AGCAGGGGGACTACCACGCCCACCTCCGCCGTCTCGTCTCCCGCGCCTTCTCCCCGAGGCCATCCGCGGCTCCGTCCCC
2 TsABA8'OH-A             77.9%  99.9%    AGCAGGGGGACTACCACGCCCACCTCCGCCGTCTCGTCTCCCGCGCCTTCTCCCCGAGGCCATCCGCGGCTCCGTCCCC
3 TsABA8'OH-B             76.2%  94.3%    AGCAGGGGGACTACCACGCTCACCTCCGCCGTCTGGTCTCCCGCGCCTTCTCCCCGAGGCCATCCGCGGCTCCGTCCCC
4 TsABA8'OH-R             77.0%  92.3%    AGCAGGGGGACTACCACGCCCACCTCCGCCGCCTCGTCTCCCGCGCCTTCTCCCCGAGGCCATCCGCGGCTCCGTCCCC

                          cov    pid 481           .         5         .         .         .         .         :         . 560
1 AB714574.1[genA_ex1-3] 100.0% 100.0%    GCCATCGAGGCTATCGCCCTCCGCTCCCTCGGCTCCTGGGAAGACCTGCAAGTCAACACCTTCCAGGAGATGAAGACTgt
2 TsABA8'OH-A             77.9%  99.9%    GCCATCGAGGCTATCGCCCTCCGCTCCCTCGGCTCCTGGGAAGACCTGCAAGTCAACACCTTCCAGGAGATGAAGACTgt
3 TsABA8'OH-B             76.2%  94.3%    GCCATCGAGGCTATCGCCCTCCGCTCCCTCGGCTCCTGGGAAGACCTTCAAGTCAACACCTTCCAAGAGATGAAGACTgt
4 TsABA8'OH-R             77.0%  92.3%    GCCATCGAGGCCATCGCCCTCCGCTCCCTCGCCTCCTGGGAGGACCTGCAGGTCAACACCTTCCAAGAGATGAAGACTgt
                                                                                                   ABA2_ngsR

                          cov    pid 561           .         .         .         6         .         .         .         . 640
1 AB714574.1[genA_ex1-3] 100.0% 100.0%    gagtgcttcttcttcttcctcctcttccattccccgccacttgctctgctttcctctgctctgctctagtgctaaatgat
2 TsABA8'OH-A             77.9%  99.9%    gagtgcttcttcttcttcctcctcttccattccccgccacttgctctgctttcctctgctctgctctagtgctaaatgat
3 TsABA8'OH-B             76.2%  94.3%    gagtgcttcttcctcttcca-------------ttcccacttgctctgcttccctctgctctgctctactgctaaataat
4 TsABA8'OH-R             77.0%  92.3%    gagtgcctcttcttcttcttccattcccact----------tgctctgctttcctctgctctgctctagtgctgaatgat

                          cov    pid 641           :         .         .         .         .         7         .         . 720
1 AB714574.1[genA_ex1-3] 100.0% 100.0%    tgg----agctcgaggctgatcg-----ttgtgttggtgttatggcgcagTACGCTCTGAATGTGGCATTGCTGTCCATC
2 TsABA8'OH-A             77.9%  99.9%    tgg----agctcgaggctgatcg-----ttgtgttggtgttatggcgcagTACGCTCTGAATGTGGCATTGCTGTCCATC
3 TsABA8'OH-B             76.2%  94.3%    tgg----agcttgagactgatcc-----ttctgttggtgtcgtggcgcagTACGCTCTGAATGTGGCATTGCTGTCCATC
4 TsABA8'OH-R             77.0%  92.3%    tgattggagcttgaggctgatggtttctttggtgtcatggcgggcgcagTACGCTCTGAATGTGGCATTGCTGTCCATC

                          cov    pid 721           .         .         :         .         .         .         8 800
1 AB714574.1[genA_ex1-3] 100.0% 100.0%    TTCGGGGAGGAGGAGATGCAGTACATCGAGGAGCTGAAGCAGTGCTACCTGACGCTGGAGAAGGGGTACAACTCGATGCC
2 TsABA8'OH-A             77.9%  99.9%    TTCGGGGAGGAGGAGATGCAGTACATCGAGGAGCTGAAGCAGTGCTACCTGACGCTGGAGAAGGGGTACAACTCGATGCC
3 TsABA8'OH-B             76.2%  94.3%    TTCGGGGAGGAGGAGATGCAGTACATCGAGGAGCTGAAGCAGTGCTACCTGACGCTGGAGAAGGGGTATAACTCGATGCC
4 TsABA8'OH-R             77.0%  92.3%    TTCGGCGAGGAGGAGATGCAGTACATCGAGGAGCTGAAGCAGTGCTACCTGACACTGGAGAAGGGGTACAACTCGATGCC

                          cov    pid 801           .         .         .         .         .         :         .         . 880
1 AB714574.1[genA_ex1-3] 100.0% 100.0%    GGTGAACCTGCCGGGCACGCTGTTCCACAAGGCCATGAAGGCCCGGAAGCGGCTGGGCGCCATTGTGGCCCACATCATCT
2 TsABA8'OH-A             77.9%  99.9%    GGTGAACCTGCCGGGCACGCTGTTCCACAAGGCCATGAAGGCCCGGAAGCGGCTGGGCGCCATTGTGGCCCACATCATCT
3 TsABA8'OH-B             76.2%  94.3%    GGTGAACCTGCCGGGCACGCTGTTCCACAAGGCCATGAAGGCCCGGAAGCGGCTGGGCGCCATTGTGGCCCACATCATCT
4 TsABA8'OH-R             77.0%  92.3%    GGTGAACCTGCCGGGCACGCTGTTCCACAAGGCCATGAAGGCCCGCAAGCGGCTGGGCGCCATTGTGGCCCACATCATCT
                                                                                              ABA3_ngsF

                          cov    pid 881           .         9         .         .         .         .         .         . 960
1 AB714574.1[genA_ex1-3] 100.0% 100.0%    CGGCCCGGCGCGAGCGCGAGCGCGGGAGCGACCTCCTGGGCTCCTTCATGGACGGCCGCGAGGCGCTCACCGACGACCAG
2 TsABA8'OH-A             77.9%  99.9%    CGGCCCGGCGCGAGCGCGAGCGCGGGAGCGACCTCCTGGGCTCCTTCATGGACGGCCGCGAGGCGCTCACAGACGACCAG
3 TsABA8'OH-B             76.2%  94.3%    CGGCCCGGCGCGAGCGTGAGCGCGGGAGCGACCTCCTGGGCTCCTTCATGGACGGCCGCGAGGCGCTCACCGACGACCAG
4 TsABA8'OH-R             77.0%  92.3%    CGGCCCGGCGCGAGCGGGAGCGCGGGAGCGACCTCCTGGGCTCCTTCATGGACGGCCGCGAGGCGCTCACCGACGACCAG

                          cov    pid 961           .         .         .         0         .         .         . 1040
1 AB714574.1[genA_ex1-3] 100.0% 100.0%    ATCGCCGACAACGCCATCGGCGTCATCTTCGCCGCGCGCGACACCACCGCCAGCGTGCTCACGTGGATGGTCAAGTTCCT
2 TsABA8'OH-A             77.9%  99.9%    ATCGCCGACAACGCCATCGGCGTCATCTTCGCCGCGCGCGACACCACCGCCAGCGTGCTCACGTGGATGGTCAAGTTCCT
3 TsABA8'OH-B             76.2%  94.3%    ATCGCCGACAACGCCATCGGCGTCATCTTCGCCGCGCGCGACACCACCGCCAGCGTGCTCACGTGGATGGTCAAGTTCCT
4 TsABA8'OH-R             77.0%  92.3%    ATCGCCGACAACGCCATCGGCGTCATCTTCGCCGCGCGGGACACCACCGCCAGCGTGCTCACGTGGATGGTCAAGTTCCT
                                                                                        PAM      ABA/2/323
```

```
                            cov    pid 1041          :         .         .         .         .         1         .         . 1120
1 AB714574.1[genA_ex1-3] 100.0% 100.0%       CGGCGACAACCCCGCCGTCCTCAAAGCCGTCACCgtaagtcgccgtcatttcatgaacccagctgacccgctaggcaccc
2 TsABA8'OH-A              77.9%  99.9%       CGGCGACAACCCCGCCGTCCTCAAAGCCGTCACCgtaagtcgccgtcatttcatgaacccagctgacccgctaggcaccc
3 TsABA8'OH-B              76.2%  94.3%       CGGCGACAACCCCGCCGTGCTCAAAGCCGTCACCgtaagtcgccatcaa---------ccagctgacccgcttggtaccc
4 TsABA8'OH-R              77.0%  92.3%       CGGCGACAACCCCGCCGTCCTCAAAGCCGTCACCgtaagtcgccatcatttcatgaacca-gctgacccgcttggtgccc

                            cov    pid 1121          .         .         :         .         .         .         .         2 1200
1 AB714574.1[genA_ex1-3] 100.0% 100.0%       gatcgaaaagcagcgactgaccc---gttcatcatacaatcaacagGAAGAGCACGCCGAGATCGCGAGGGAGAAGGCGT
2 TsABA8'OH-A              77.9%  99.9%       gatcgaaaagcagcgactgaccc---gttcatcatacaatcaacagGAAGAGCACGCCGAGATCGCGAGGGAGAAGGCGT
3 TsABA8'OH-B              76.2%  94.3%       gatcgaaaagcagcgactgaccc---gtgcatccaacaattaacagGAAGAGCATGCCGAGATCGCGAGGGAGAAGGCGT
4 TsABA8'OH-R              77.0%  92.3%       gatcgaaaggcagccactgacctgacctgtattgaacaaataacagGAAGAGCACGCTGAGATCGCGAGGGAGAAGGCGT
                                                                                   Aex3R/Bex3R/Rex3R          ABA3_ngsR
                            cov    pid 1201          .         .         .         .         :         .         .         . 1280
1 AB714574.1[genA_ex1-3] 100.0% 100.0%       TGTCCGGCGAGGCGCTGTCGTGGGCCGACACGCGGCGGATGCGGTTGACGGGCCGGGTGATCCAGGAGACGATGCGGGTG
2 TsABA8'OH-A              77.9%  99.9%       TGTCCGGCGAGGCGCTGTCGTGGGCCGACACGCGGCGGATGCGGTTGACGGGCCGGGTGATCCAGGAGACGATGCGGGTG
3 TsABA8'OH-B              76.2%  94.3%       TGTCCGGCGAGCCGCTGTCGTGGGCCGACACGCGGCGGATGCGGATGACGGGCCGGGTGATCCAGGAGACGATGCGGGTG
4 TsABA8'OH-R              77.0%  92.3%       TGTCCGGCGAGCCGCTGTCGTGGGCCGACACGCGGCGGATGCGGGTGACGGGGCGGGTGATCCAGGAGACGATGCGGGTG

                            cov    pid 1281          .         3         .         .         .    ] 1335
1 AB714574.1[genA_ex1-3] 100.0% 100.0%       GCGTCCATCCTCTCCTTCACCTTCAGGGAGGCCGTGGAGGACGTGGAGTACCAAG
2 TsABA8'OH-A              77.9%  99.9%       G------------------------------------------------------
                                                      ABA_uR
3 TsABA8'OH-B              76.2%  94.3%       G------------------------------------------------------
4 TsABA8'OH-R              77.0%  92.3%       G------------------------------------------------------
```

Figure S1. TsABA8'OH partial sequences of Triticale cv. Bogo (NCBI MW538321-MW538323) generated with Clustal Omega and visualized with MView on-line tools [47-48]. Sequences were aligned to common wheat genone A reference sequence of ABA 8'-hydroxylase. Primers and gRNAs used in this study were marked with black boxes. Notice NSP in R genome sequence of ABA/1/364 and site for introduced mismatches in genome specific primers Aex3R, Bex3R and Rex3R (marked with red boxes, compare to Table S1).
