# Supplementary material for: Functional Validation of cas9/GuideRNA Constructs for Site-Directed Mutagenesis of Triticale ABA8′OH1 loci
Source: Int J Mol Sci. 2021 Jun 29;22(13):7038. doi: 10.3390/ijms22137038 (PMC8269138; doi:10.3390/ijms22137038)
Supplement: Supplementary file 1 [file ijms-22-07038-s001.zip › ijms-1240053-supplementary/Figure S2.pdf]

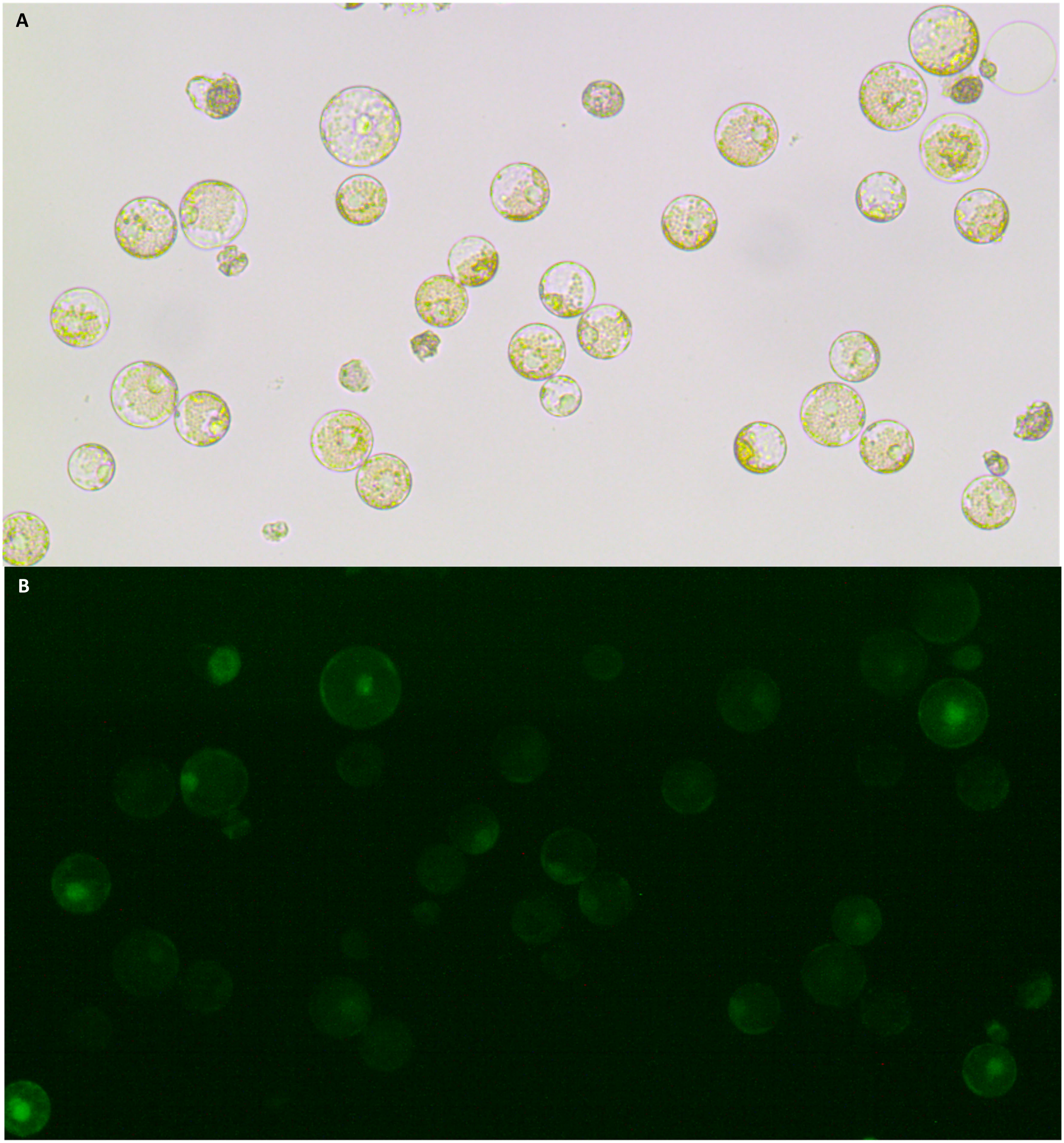

Figure S2: Population of triticale mesophyll protoplasts 48h after transfection with CRISPR/Cas9 coding plasmid. Brightfield (A) and fluorescence (B) observations of green fluorescence protein (GFP) were used to estimate transfection efficiency. Magnification: 200x.
