# Supplementary material for: Functional Validation of cas9/GuideRNA Constructs for Site-Directed Mutagenesis of Triticale ABA8′OH1 loci
Source: Int J Mol Sci. 2021 Jun 29;22(13):7038. doi: 10.3390/ijms22137038 (PMC8269138; doi:10.3390/ijms22137038)
Supplement: Supplementary file 1 [file ijms-22-07038-s001.zip › ijms-1240053-supplementary/Figure S3.pptx]

## Slide 1
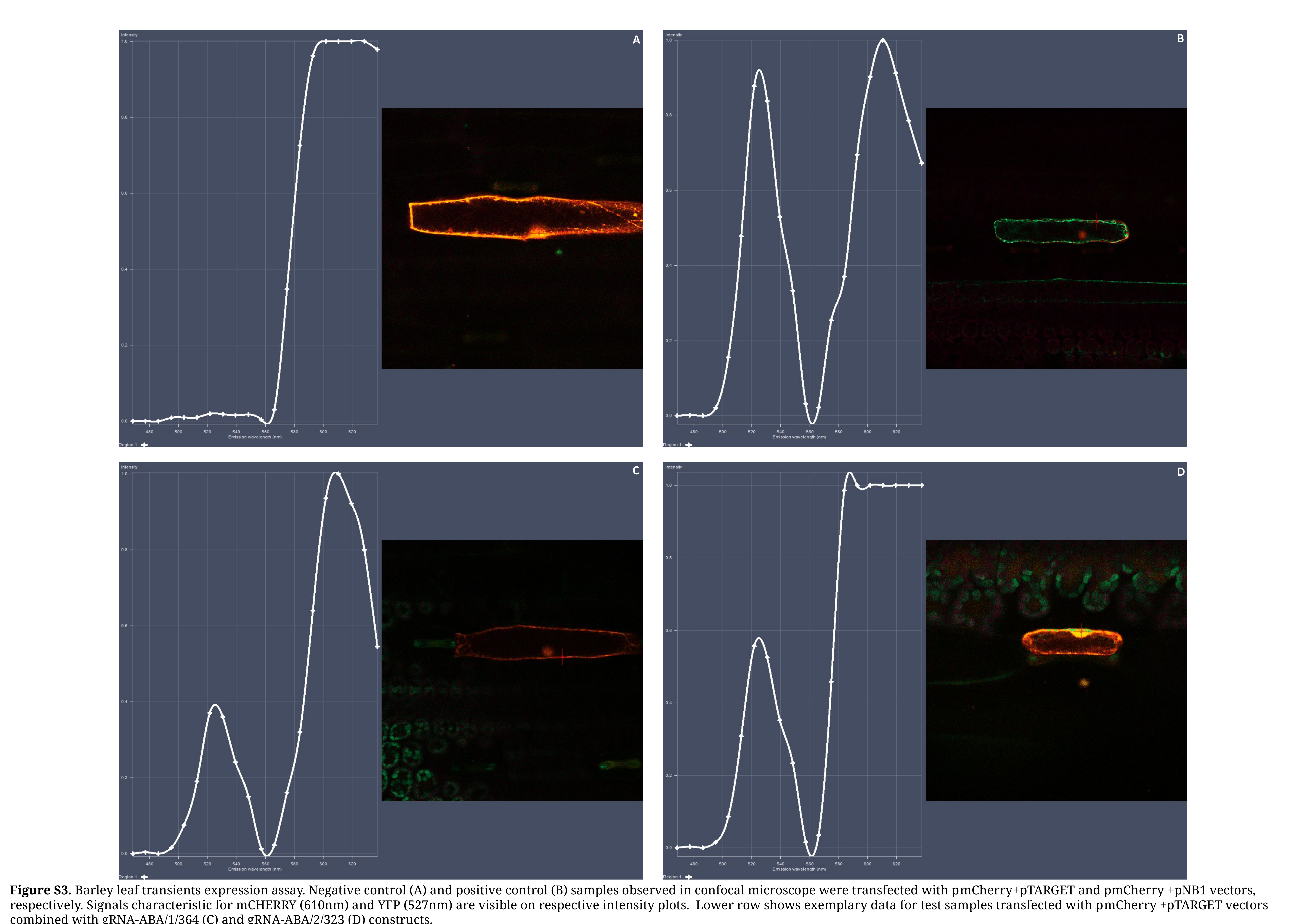

B
A
C
D
Figure S3. Barley leaf transients expression assay. Negative control (A) and positive control (B) samples observed in confocal microscope were transfected with pmCherry+pTARGET and pmCherry +pNB1 vectors, respectively. Signals characteristic for mCHERRY (610nm) and YFP (527nm) are visible on respective intensity plots. Lower row shows exemplary data for test samples transfected with pmCherry +pTARGET vectors combined with gRNA-ABA/1/364 (C) and gRNA-ABA/2/323 (D) constructs.
