# Supplementary material for: Functional Validation of cas9/GuideRNA Constructs for Site-Directed Mutagenesis of Triticale ABA8′OH1 loci
Source: Int J Mol Sci. 2021 Jun 29;22(13):7038. doi: 10.3390/ijms22137038 (PMC8269138; doi:10.3390/ijms22137038)
Supplement: Supplementary file 1 [file ijms-22-07038-s001.zip › ijms-1240053-supplementary/Table S1.pdf]

Table S1. Primers used for PCR amplifications and sequencing. Mismatches are indicated in red. Underlined are the sequences used for NGS library construction.

Name	Sequence (5′-3′)	Application
ABA_uF	TACTCCTCCAAGAACCCCAAC	Pair of common primers used for amplification of TsABA8′OH sequences prior to cloning and Sanger sequencing
ABA_uR	AAGGTGAAGGAGAGGATGGA	
ABA2_ngsF	TCGTCGGCAGCGTCAGATGTGTATAAGAGACAGAAGAACCCCAACGTCTTCTT	Pair of common primers used for amplification of sequences targeted by gRNA-ABA/1/364 prior to deep sequencing. Library construction adapters were underlined.
ABA2_ngsR	GTCTCGTGGGCTCGGAGATGTGTATAAGAGACAGGCACTCACAGTCTTCATCTC	
ABA3_ngsF	TCGTCGGCAGCGTCAGATGTGTATAAGAGACAGATTGTGGCCCACATCATCTC	Pair of common primers used for amplification of sequences targeted by gRNA-ABA/2/323 prior to deep sequencing. Library construction adapters were underlined.
ABA3_ngsR	GTCTCGTGGGCTCGGAGATGTGTATAAGAGACAGCCTTCTCCCTCGCGATCTC	
RABex1F	GGCCCATCTTCAAGACGCA	Common forward primer used in pairs with one of three genome specific primers in T7EI assay.
Aex3R	GGCGTGCTCTTCCTGTTGATTGAAT	Genome A-specific primer used for PCR amplification in T7EI assay. Notice additional mismatch introduced to improve its specificity in third position to 3′ end (marked red).
Bex3R	GGCATGCTCTTCCTGTTAATTGATG	Genome B-specific primer used for PCR amplification in T7EI assay. Notice additional mismatch introduced to improve its specificity in third position to 3′ end (marked red).
Rex3R	AGCGTGCTCTTCCTGTTATTTGATC	Genome R-specific primer used for PCR amplification in T7EI assay. Notice additional mismatch introduced to improve its specificity in third position to 3′ end (marked red).
