# Supplementary material for: Functional Validation of cas9/GuideRNA Constructs for Site-Directed Mutagenesis of Triticale ABA8′OH1 loci
Source: Int J Mol Sci. 2021 Jun 29;22(13):7038. doi: 10.3390/ijms22137038 (PMC8269138; doi:10.3390/ijms22137038)
Supplement: Supplementary file 1 [file ijms-22-07038-s001.zip › ijms-1240053-supplementary/Table S2.pdf]

Table S2: Mutation frequencies in Triticale protoplasts acquired by deep-sequencing of short reads. Type and size of mutation generated by individual constructs was quantified as a percentage of the total construct efficiency.

			gRNA-ABA/1/364			gRNA-ABA/1/364+ TREX2			gRNA-ABA/2/323			gRNA-ABA/2/323+ TREX2		
			genome			genome			genome			genome		
			A	B	R	A	B	R	A	B	R	A	B	R
Mutation type frequency [%]	1 bp	del	0.37	0.25	0.59	0.09	0.04	0.00	36.23	39.06	41.78	0.89	0.15	0.04
		ins	0.03	0.04	0.07	0.00	0.02	0.00	19.29	26.31	21.60	0.00	0.00	0.00
		sub	3.01	0.17	1.23	0.01	0.15	0.00	2.67	1.23	0.38	0.09	0.30	0.00
	2-10 bp	del	28.92	30.46	28.07	14.04	12.00	11.24	32.65	28.70	35.48	14.42	15.19	12.31
		ins	0.00	0.00	0.00	0.02	0.00	0.00	0.00	0.23	0.00	0.00	0.00	0.00
		sub	0.08	0.17	0.62	0.01	0.00	0.00	0.00	0.00	0.00	0.05	0.00	0.00
	11-99 bp	del	66.08	66.30	68.06	83.88	85.01	86.37	2.82	2.46	0.66	82.60	83.64	84.09
		ins	0.55	0.66	0.13	0.02	0.06	0.00	6.34	2.01	0.09	0.00	0.00	0.00
		sub	0.06	0.92	0.00	0.01	1.80	0.15	0.00	0.00	0.00	0.00	0.00	0.00
	>100 bp	del	0.28	0.04	0.57	1.39	0.89	2.03	0.00	0.00	0.00	1.60	0.41	3.49
		ins	0.07	0.00	0.04	0.01	0.00	0.01	0.00	0.00	0.00	0.00	0.00	0.00
		sub	0.00	0.00	0.00	0.00	0.00	0.00	0.00	0.00	0.00	0.00	0.00	0.00
	Complex modifications		0.54	0.99	0.62	0.43	0.04	0.19	0.00	0.00	0.00	0.34	0.32	0.07
